# Physician attitudes and treatment patterns for pancreatic cancer

**DOI:** 10.1186/1477-7819-9-21

**Published:** 2011-02-11

**Authors:** Jarret Woodmass, Jeremy Lipschitz, Andrew McKay

**Affiliations:** 1 Faculty of Medicine, University of Manitoba, Winnipeg, MB, R3E 3P5, Canada; 2 Department of Surgery, Health Sciences Centre, University of Manitoba, Winnipeg, MB, R3A 1R9, Canada; 3 Department of Community Health Sciences, University of Manitoba, Winnipeg, MB, R3E 0W3, Canada; 4 Epidemiology and Cancer Registry, CancerCare Manitoba, Winnipeg, MB, R3E 0V9, Canada

## Abstract

**Background:**

Surgery appears to be an underutilized treatment option for pancreatic cancer. Nihilistic physician attitudes may be partly responsible. The study objectives were to analyze physician attitudes towards this disease and determine treatment patterns and outcomes including rates of surgical referral.

**Methods:**

A survey was administered to 420 physicians in Manitoba to document general knowledge and attitudes. Population based administrative data was accessed for all patients diagnosed with pancreatic cancer between 2004 and 2006 to examine treatment patterns and outcomes.

**Results:**

181 physicians responded to the survey. Most (73%) believed that surgical resection was worthwhile. Of the 413 Manitobans diagnosed with pancreatic cancer, only 11% underwent an attempt at surgical resection. There were 124 patients with stage I or II disease (i.e. potentially resectable), 85 of these patients received no treatment and 39% were not referred to a surgeon. These patients were older than those referred, but did not have more comorbidities.

**Conclusion:**

Most physicians were insightfully aware of both the survival benefit and potential risks of surgical resection. However, some did overestimate the surgical mortality and underestimate the associated survival benefit. Although advanced age may justly account for some of the patients not receiving a referral, it is reasonable to assume that nihilistic physician attitudes is contributing to the apparent underutilization of surgery for pancreatic cancer. Efforts should be made to ensure that eligible patients are at least offered surgery as a potential treatment option.

## Background

Pancreatic cancer is an aggressive malignancy which portends a very poor prognosis. In 2009 it is estimated that there will be 3900 new cases of pancreatic cancer in Canada, with an equal number of deaths from the disease. This makes it the 5^th ^leading cause of cancer-related death[1]. Currently, the treatment offering the best chance for disease-free long-term survival is surgical resection of the tumor[2]. However, it appears that only 20-35% of the potentially resectable patients are undergoing surgery[2-5]. One potential reason for the low rate of surgical resection may be the nihilistic view held by many physicians towards pancreatic resection. Throughout the 1970's many authors had suggested abandoning the use of surgical resection altogether as a treatment for pancreatic cancer due to an operative mortality rate in excess of 20%[5-8] and a variable 5-year survival rate averaging approximately 7%[7].

Major pancreatic surgery is now safer than it has ever been, and there is good evidence to suggest a survival benefit[2]. High volume centers now routinely report mortality rates between 1% and 4%[9] and five-year survival rates exceeding 15%[10-12]. Although there may exist some controversy about the definition of "high volume", a cut-off of 11 or more pancreatectomies per year is often used to define a high volume center[13]. Despite these improvements, in a large population-based study in the United States Bilimoria et al found that even after accounting for severe comorbidities, advanced age and patient refusal, 38% of patients with stage I disease were not even offered surgery[3]. It cannot be overlooked that there is still a relatively poor prognosis for patients with pancreatic cancer even with pancreatectomy, and that the surgery carries serious morbidity and mortality. However, such patients should at least have surgery presented as an available treatment option. The aim of this study was to assess physician attitudes towards pancreatic cancer in order to identify any factors that may be limiting the treatment options being made available to patients. Additionally, we assessed the current treatment patterns of pancreatic cancer in the Province of Manitoba including the rate of surgical referral, overall mortality and 3-year survival.

## Methods

### Survey

The questionnaire was designed based on the recommendations of Polgar and Thomas[14]. A pilot was administered to a focus group consisting of six physicians with varying levels of knowledge about pancreatic cancer to identify/correct any problems or ambiguity within the survey.

Two hundred of the 902 Family Physicians with an active license to practice in Manitoba were randomly selected to receive the survey. In addition, all physicians practicing in General Surgery, Internal Medicine, Gastroenterology, Radiation/Medical Oncology and Geriatrics were administered the survey, for a total of 480 physicians.

### Treatment Patterns and Outcomes

The Manitoba Cancer Registry (MCR) was used to identify all patients diagnosed with pancreatic cancer in the Province of Manitoba between January 1, 2004 and December 31, 2006. The year 2004 was chosen because this is the first year the Cancer Registry began recording detailed collaborative TNM staging data. The stop date in 2006 allowed for at least a 3-year follow-up period for each patient enabling an analysis of survival trends associated with the different treatments provided to pancreatic cancer patients. Only patients with adenocarcinoma of the pancreas were included.

The MCR is linked to several administrative databases managed by Manitoba Health including the Medical Claims Database, the Hospital Separations Abstracts, and the Manitoba Health Registry. The combined information from these databases provided regional data (eg. incidence/prevalence/mortality), demographic data (eg. age/sex/income), treatment information (eg. referral/surgery/chemotherapy) and comorbidities (up to 16 comorbid diseases). The MCR also provided detailed tumor-specific information and TNM status as derived from pathology, histology and cytology reports. The MCR uses a collaborative staging system where surgical pathology is used to determine stage when available. In cases where surgery is not performed, the stage is assigned according to the best available data including diagnostic imaging, cytology, histology, laboratory and clinical results. Each patient was staged according to the American Joint Committee on Cancer (AJCC) Staging Manual, 6^th ^ed[15]. For the purpose of analyzing the data, patients with AJCC Stage I or II disease were grouped as "Early Stage" and those with AJCC Stage III or IV disease were grouped as "Late Stage". This is because patients with Stage III or IV disease have either involvement of the superior mesenteric artery or celiac axis (Stage III) or metastatic disease (Stage IV) that is not amenable to surgical resection.

### Statistical Analysis

The physician population of most interest for the survey was the primary care physicians. Using the most conservative estimate that 50% of respondents would consider that surgical treatment of pancreatic cancer was not worthwhile it was determined that 96 survey responses would be required to be 95% confident that the true proportion was within +/- 10% of that figure (P = 0.80). Because the anticipated response rate was only 50% a total of 200 randomly selected primary care physicians were included to receive the survey. The entire population of specialists was included in the mailing list due to the small numbers of these specialists. To examine the relationship between the ordinal responses obtained from the questionnaire a Spearman's rank correlation test was utilized.

The sample of patients with pancreatic cancer used to evaluate treatment patterns was one of convenience; all available patients in the MCR for the years 2004 through 2006 were included. The anticipated sample size was 450 patients[1]. A multivariate multinomial regression model was used to examine the relationship between predictor variables and the type of treatment received. A multivariate logistic regression model was used to examine the relationship between predictor variables and whether a surgical referral was obtained. A multivariate Cox regression was used to examine the relationship between predictor variables and all-cause mortality. A p-value of 0.05 was used to define statistical significance for all analyses.

## Results

### Survey

A total of 480 physicians were included in the initial mailing, however, 60 were later excluded due to having an inaccurate mailing address (56), having retired (3), or being recently deceased (1). Of the 420 potential responders 181 questionnaires were completed for a response rate of 43%. This included 72 family physicians, 45 internists, 35 general surgeons, 10 medical/radiation oncologists, 9 gastroenterologists, and 5 geriatricians. The response rate for these subgroups ranged from 40% for internists to 64% for gastroenterologists.

**Figure 1:**
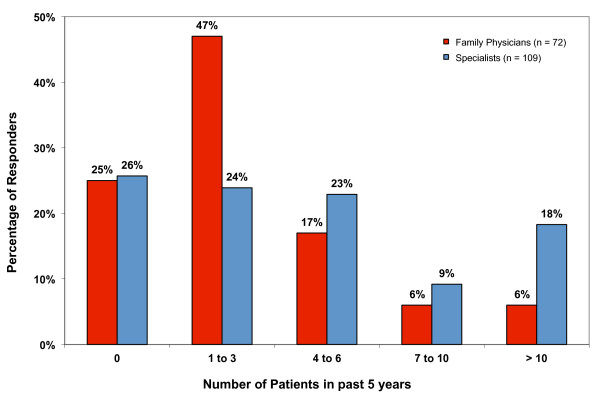
**The estimated case volume of pancreatic cancer patients treated during a 5-year time period**.

The number of pancreatic cancer patients examined by all physicians in their practice over the past 5 years ranged from zero to greater than 10 with the majority having seen either 0 (25%), 1-3 (33%), or 4-6 (20%) patients. The estimated volume of pancreatic cancer patients seen by family physicians and specialists are shown separately in Figure 1. When asked about their familiarity with the treatment of pancreatic cancer 74% of all physicians surveyed (87% of family physicians surveyed) stated that they were unsure of the difference in survival benefit expected with the different treatments available.

**Figure 2:**
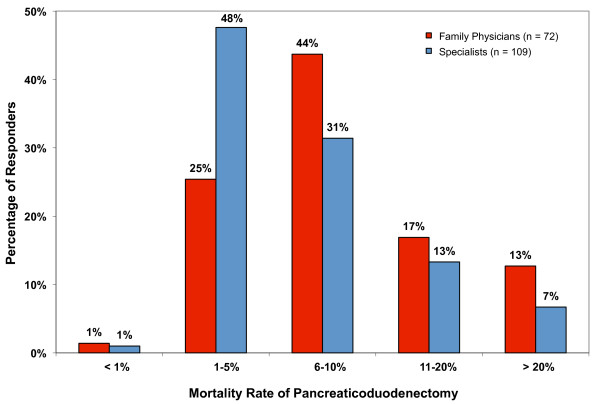
**Physician estimated mortality rates associated with the surgical resection of a pancreatic tumor**.

The perioperative mortality rate associated with pancreaticoduodenectomy (PD) estimated by physicians ranged from <1% to >20%. The most common physician response (39%) estimated a mortality rate of 1-5% percent with a similar proportion (36%) suggesting it was 6-10%. Fifteen percent estimated a mortality rate of 10-20% while 9% estimated a mortality rate >20%. The mortality rates estimated by family physicians and specialists are shown separately in Figure 2.

The majority of survey respondents (73%) considered PD to be worthwhile (Table 1 - Physician responses to statements regarding the treatment of patients with early and late stage pancreatic cancer). A greater percentage of surgeons stated that surgery was worthwhile than family physicians but this difference was not statistically significant (p = 0.12). Furthermore, a significantly greater proportion of surgeons (77%) stated that surgical resection could cure a patient of pancreatic cancer than did family physicians (41%) (p = 0.002). The majority of gastroenterologists reported surgery to be both worthwhile (89%) and a potentially curative procedure (67%). For patients with late stage disease there was discrepancy amongst physicians for the usefulness of chemotherapy and/or radiation therapy (Table 1).

**Table 1:** Physician responses to statements regarding the treatment of early and late stage pancreatic cancer.

Statements pertaining to patients with resectable disease			
	**Agree**	**Disagree**	**Neutral**
The mortality rate is too high to undergo surgery	2.3%	90%	7.6%
The associated morbidity is too high to undergo surgery	4.7%	80%	15%
Limited resources prevents patient access to surgery	12%	68%	21%
The benefit is too small to warrant surgery	5.8%	75%	19%
Surgical resection can cure a patient of pancreatic cancer	53%	22%	26%
Surgery is worth while for these patients	73%	6.4%	20%

**Statements pertaining to patients with unresectable disease**			

	**Agree**	**Disagree**	**Neutral**
The associated morbidity is too high to undergo chemotherapy	31%	38%	30%
The benefit is too small to warrant chemotherapy	38%	31%	31%
Chemotherapy can cure a patient of pancreatic cancer	1%	92%	6%
Chemotherapy is worth while for these patients	29%	27%	43%

### Treatment Patterns and Outcomes

A total of 413 patients were diagnosed with pancreatic cancer during our study period. Of these, 124 (30%) had early stage disease, 252 (61%) had late stage disease and 37 (9.0%) patients were of unknown stage. A total of 46 patients were staged surgically while the rest of the patients were staged clinically, most often with CT scans. For the 376 patients with complete staging information in the MCR, 50% had pathologic confirmation of the diagnosis.

Most patients (79%) diagnosed with pancreatic cancer did not receive any treatment. Forty-six patients (11%) underwent an attempt at curative resection, 35 (8.5%) of which were successful. There were 26 pancreaticoduodenectomies performed. An additional forty patients underwent chemotherapy and/or radiotherapy. In a multivariate multinomial regression analysis, the greatest predictor for a patient to undergo surgery was the stage of the disease with early stage patients being much more likely than late stage patients (odds ratio [OR] = 42; 95% confidence interval [CI] = 11 - 158). Patients were also more likely to undergo surgery if they were younger than 65 years of age (OR = 5.3; CI = 2.0 - 14).

Of the 124 patients diagnosed with potentially resectable (early stage) disease, 28 (23%) underwent surgical resection (5 with adjuvant chemotherapy; 1 with adjuvant radiotherapy), 6 (4.8%) had chemotherapy only, 1 (0.81%) had chemo/radiotherapy, 4 (3.2%) began the operation and were considered unresectable, and 85 (69%) received no treatment (Table 2 - Demographic outline and treatment patterns of patients diagnosed with pancreatic cancer). The only statistically significant predictor of whether patients with early stage disease underwent surgery was age. Patients younger than 65 were more likely to undergo surgery (OR = 4.6; 95% CI = 1.2 to 17).

**Table 2:** Demographic outline and treatment patterns of patients diagnosed with pancreatic cancer.

Characteristic		Stage of Disease*		
		Early (n = 124)	Late (n = 252)	Unknown (n = 37)
Treatment				
	Surgery	28 (23%)	3 (1.2%)	4 (11%)
	Other	11 (8.9%)	40 (16%)	0 (0%)
	No Treatment	85 (69%)	209 (83%)	33 (89%)
Gender				
	Male	69 (56%)	123 (49%)	18 (49%)
	Female	55 (44%)	129 (51%)	19 (51%)
Age				
	64 and younger	36 (29%)	85 (34%)	4 (11%)
	65 and older	88 (71%)	167 (66%)	33 (89%)
Charlson Comorbidity				
	2 or less	71 (57%)	63 (25%)	23 (62%)
	3 or more	50 (40%)	181 (72%)	12 (32%)
	Missing	3 (2.4%)	8 (3.2%)	2 (5.4%)
Residence				
	Urban	80 (65%)	159 (63)	23 (62%)
	Rural	43 (35%)	91 (36%)	13 (35%)
	Missing	1 (0.81%)	2 (0.79%)	1 (2.7%)

Of the early stage patients, 35 (28%) were not referred to a pancreatic surgeon. Additionally, of the 85 early stage patients who did not receive treatment, 33 (39%) did not receive surgical consultation. On univariate analysis, patients who were not referred to a surgeon were older than those who were referred (mean age 81 compared to 68; p < 0.0001) and had less comorbidity than those who were referred (Wilcoxon rank; p = 0.006). On multivariate analysis, only younger age remained a significant predictor of surgical referral (OR = 5.57; 95% CI = 1.5 - 21).

**Figure 3:**
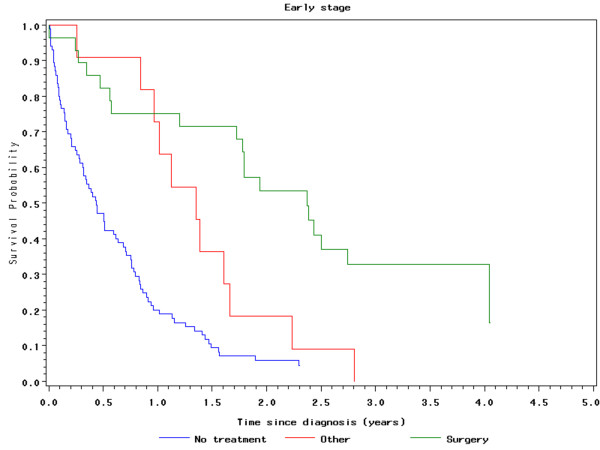
**Survival of early stage pancreatic cancer patients following the utilization of different treatment modalities**.

For all patients who underwent surgery (those with early and late stage disease), the operative 30-, 60- and 90- day mortality rates were 2.9%%, 8.1% and 11.4% respectively. Patients with early stage disease who underwent surgery (n = 28) had a median overall survival of 28 months and a 3-year survival of 33%. Early stage patients who did not have surgery had a median survival of 6.1 months and a 3-year survival of 3.8% (Figure 3 - Survival of early stage pancreatic cancer patients following the utilization of different treatment modalities). Significant predictors of survival for early stage pancreatic cancer patients included surgical intervention (hazard ratio [HR] = 0.24; 95% CI = 0.14 - 0.42), "other" treatment (HR = 0.51; 95% CI = 0.27 - 0.95) and a Charlson comorbidity score of 2 or less (HR = 0.56; 95% CI = 0. 37 to 0.85)[16].

## Discussion

Many physicians may not be fully aware of the recent improvements in the surgical treatment of pancreatic cancer because this is a disease that most physicians encounter rarely. In our study 88% of family physicians estimated seeing six or less pancreatic cancer patients in their practice over the past 5 years and 87% stated that they were unsure of the differences in survival expected with the different treatments available. Despite this, most physicians surveyed (73%) believed that surgery was a worthwhile treatment for patients with potentially resectable disease (Table 1). While this was most notable amongst the Gastroenterologists who responded (89%), the limited number (n = 9) of such participants in this study was too small to draw any firm conclusions. We did, however, identify a couple areas of concern amongst the responses. Firstly, a large number of physicians continue to overestimate the mortality rate associated with PD. Recent studies indicate that the perioperative mortality in high-volume centers varies from 1-4%[9]. However, in our study only 27% of family physicians estimated the mortality rate to be 5% or less. Even more concerning was that nearly a third estimated the mortality rate to exceed 10% (Figure 2). Secondly, only 41% of family physicians stated that surgical resection could potentially cure a patient from pancreatic cancer. This is in contrast to the 77% of the surgeons responding. These findings suggest there is a small, but important, proportion of the referral base who overestimate the mortality of surgery for pancreatic cancer and underestimate the benefit. This negative view of surgery may be limiting the options presented to patients.

When examining the treatment and referral patterns of patients with pancreatic cancer we found that a significant proportion (39%) of the early stage (potentially resectable) pancreatic cancer patients were not even referred to a pancreatic surgeon for consultation. Age less than 65 was the only statistically significant predictor of being referred to a surgeon on multivariate analysis. However, advanced age alone should not necessarily preclude a patient from having a surgical consultation. Several studies have demonstrated the safety of major pancreatic surgery in selected elderly patients, including patients greater than 80 years of age[17,18]. Furthermore, while only significant on univariate analysis, the patients who were not referred to a surgeon actually had less comorbidity than those who were. This suggests that in many cases the decision not to undertake surgical referral was not because of prohibitive comorbidity, and it is reasonable to assume that negative physician attitudes towards this disease played a role.

Using the National Cancer Database, which is a population-based database containing information on over 75% of the cancers diagnosed in the United States, Bilimoria et al also found that the proportion of patients with potentially resectable pancreatic cancer who were not offered surgery is quite high at 38%, even after controlling for advanced age, prohibitive comorbidity and patient refusal[3]. In that study, patients who were not offered surgery were older, were black, had lower income, had less education, did not have private insurance, had a tumor in the head or body of the pancreas, or were seen at a low volume or community hospital. In Manitoba, all patients have universal health coverage and all pancreatic surgery is performed in tertiary care centers. Therefore the issues of private insurance and treatment in community hospitals would not apply to this study. Perhaps this explains why the proportion of early stage patients who were not offered surgery is slightly lower in this study compared to theirs (28% versus 39%). Nevertheless, there remains a high proportion of patients with potentially resectable pancreatic cancer who are not being referred to a surgeon.

For the entire population of patients with pancreatic cancer in Manitoba, the overall operative rate for patients with Stage I or II disease was only 23% which is consistent with other reports[2,3]. The 3-year survival rate and median overall survival of these patients was 29 months and 33% in comparison to 6.1 months and 3.8% for similar patients who did not undergo surgery. These results are also similar to previous literature and support the opinion that surgery may be an underutilized treatment option for this disease[3-5].

There are several limitations to this study. The low survey response rate of 43% is problematic, but the sample size was designed based on a 50% response rate. Although every effort was made to develop a fair and valid survey, response bias must be considered. Due to confidentiality regulations this study was unable to link each physician's questionnaire responses with their clinical treatment patterns. This would have allowed us to determine if the physicians that maintain a nihilistic view of pancreatic cancer are the same physicians not referring their patients to see a pancreatic surgeon. Although we were able to determine the proportion of patients who were not referred to a pancreatic surgeon, the MCR does not record the exact reasons why. Also, the patient analysis was based on a relatively small sample size which did not carry sufficient power to verify some of the trends observed. However, at the time of the study the MCR was the only registry in Canada to record detailed TNM staging information. Therefore, this study provided a unique and important opportunity to assess the disease in Canada.

## Conclusion

Most physicians were insightfully aware of both the survival benefit and potential risks of surgical resection and reported it to be worthwhile. However, some physicians continue to overestimate the surgical mortality and underestimate the survival benefit associated with pancreaticoduodenectomy. Although advanced age may justly account for some of the patients not receiving a referral, it is reasonable to assume that the influence of nihilistic physician attitudes is contributing to the apparent underutilization of surgery.

## Competing interests

The authors declare that they have no competing interests.

## Authors' contributions

all authors have read and approved the manuscript.

JW - Design, Questionnaire Development, Data Collection, Data Analysis, Drafted manuscript, Manuscript Review.

JL - Conception, Design, Drafted Manuscript, Manuscript Review.

AM - Conception, Design, Questionnaire Development, Data Analysis, Drafted Manuscript, Manuscript Review.
